# NSun2 promotes cell migration through methylating autotaxin mRNA

**DOI:** 10.1074/jbc.RA119.012009

**Published:** 2021-01-13

**Authors:** Xin Xu, Yihua Zhang, Junjie Zhang, Xiaotian Zhang

**Affiliations:** 1 The Key Laboratory of Cell Proliferation and Regulation Biology, Ministry of Education, Institute of Cell Biology, College of Life Sciences, Beijing Normal University, Beijing, China; 2 Academy of Plateau Science and Sustainability, People's Government of Qinghai Province & Beijing Normal University, Xining, China

**Keywords:** NSun2, RNA methyltransferase, autotaxin, mRNA methylation, cell migration, RNA methylation, gene regulation, cancer, RNA-methyltransferase

## Abstract

NSun2 is an RNA methyltransferase introducing 5-methylcytosine into tRNAs, mRNAs, and noncoding RNAs, thereby influencing the levels or function of these RNAs. Autotaxin (ATX) is a secreted glycoprotein and is recognized as a key factor in converting lysophosphatidylcholine into lysophosphatidic acid (LPA). The ATX-LPA axis exerts multiple biological effects in cell survival, migration, proliferation, and differentiation. Here, we show that NSun2 is involved in the regulation of cell migration through methylating ATX mRNA. In the human glioma cell line U87, knockdown of NSun2 decreased ATX protein levels, whereas overexpression of NSun2 elevated ATX protein levels. However, neither overexpression nor knockdown of NSun2 altered ATX mRNA levels. Further studies revealed that NSun2 methylated the 3′-UTR of ATX mRNA at cytosine 2756 *in vitro* and *in vivo*. Methylation by NSun2 enhanced ATX mRNA translation. In addition, NSun2-mediated 5-methylcytosine methylation promoted the export of ATX mRNA from nucleus to cytoplasm in an ALYREF-dependent manner. Knockdown of NSun2 suppressed the migration of U87 cells, which was rescued by the addition of LPA. In summary, we identify NSun2-mediated methylation of ATX mRNA as a novel mechanism in the regulation of ATX.

mRNA methylation is an important modification type in physiological and pathological processes. NSun2, also called myc-induced SUN domain-containing protein, belongs to the NOP2/Sun domain family. As a major 5-methylcytosine (m^5^C) methyltransferase, NSun2 was initially considered to be a typical tRNA methyltransferase. Recently, increasing evidences suggest that NSun2-mediated m^5^C methylation extensively occur in mRNAs and noncoding RNAs (1, 2, 3).

It has been reported that mRNA methylation by NSun2 has vital functions in cell senescence, cell differentiation, and proliferation (4, 5, 6, 7). NSun2-mediated mRNA methylation enhances the translation of CDK1, E2F3, and p21 proteins to promote cell proliferation (8, 9). NSun2 represses the expression of p27 through methylation of p27 5′′UTR to accelerate cell growth (10). The expression of NSun2 is closely associated with tumor development and prognosis. NSun2 is highly expressed in head and neck squamous carcinoma, and the patients with high levels of NSun2 in tumor tissue have a shorter survival time (11). NSun2 overexpression is also related to the metastatic progression in human breast cancer (12). In addition, it has been reported that NSun2 and YBX1 jointly promote human urothelial carcinoma of the bladder through stabilizing HDGF mRNA (13). However, the mechanisms of NSun2-mediated mRNA methylation in tumor development remain to be further studied.

Autotaxin (ATX), also known as ectonucleotide pyrophosphatase/phosphodiesterase 2 (ENPP2), is a secreted glycoprotein that can convert lysophosphatidylcholine into lysophosphatidic acid (LPA), functioning as the major enzyme for extracellular LPA production (14). LPA is a potent bioactive lipid that is capable of activating different signal pathways through interacting with the LPA receptors on cell surface. LPA can regulate a broad range of cell functions, such as cell survival, proliferation, and migration. The ATX-LPA axis plays an important role in cancer development, especially in cancer cell migration (15, 16).

ATX has been identified as one of the top 40 high-expression genes in metastatic cancer (17). Inflammatory factors such as tumor necrosis factor α, IL-6, IL-8, and vascular endothelial growth factor upregulate the expression of ATX and promote the production of LPA in hepatocellular carcinoma (18, 19, 20). Regulation of ATX expression at the level of transcription has been intensively reported. For examples, ATX can be regulated at the transcriptional level by transcriptional factors Stat3, AP-1, and SP (21, 22, 23), as well as histone deacetylases (24). However, whether mRNA methylation is involved in the regulation of ATX is still unknown.

In the present study, we show that RNA methyltransferase NSun2 catalyzes the methylation of ATX mRNA 3′UTR at C2756. Methylation by NSun2 promotes the export of ATX mRNA from nucleus to cytoplasm in an ALYREF-dependent manner and enhances the translation of ATX. This NSun2-ATX regulatory process affects cancer cell migration.

## Results

### NSun2 regulates ATX expression

**Figure 1:**
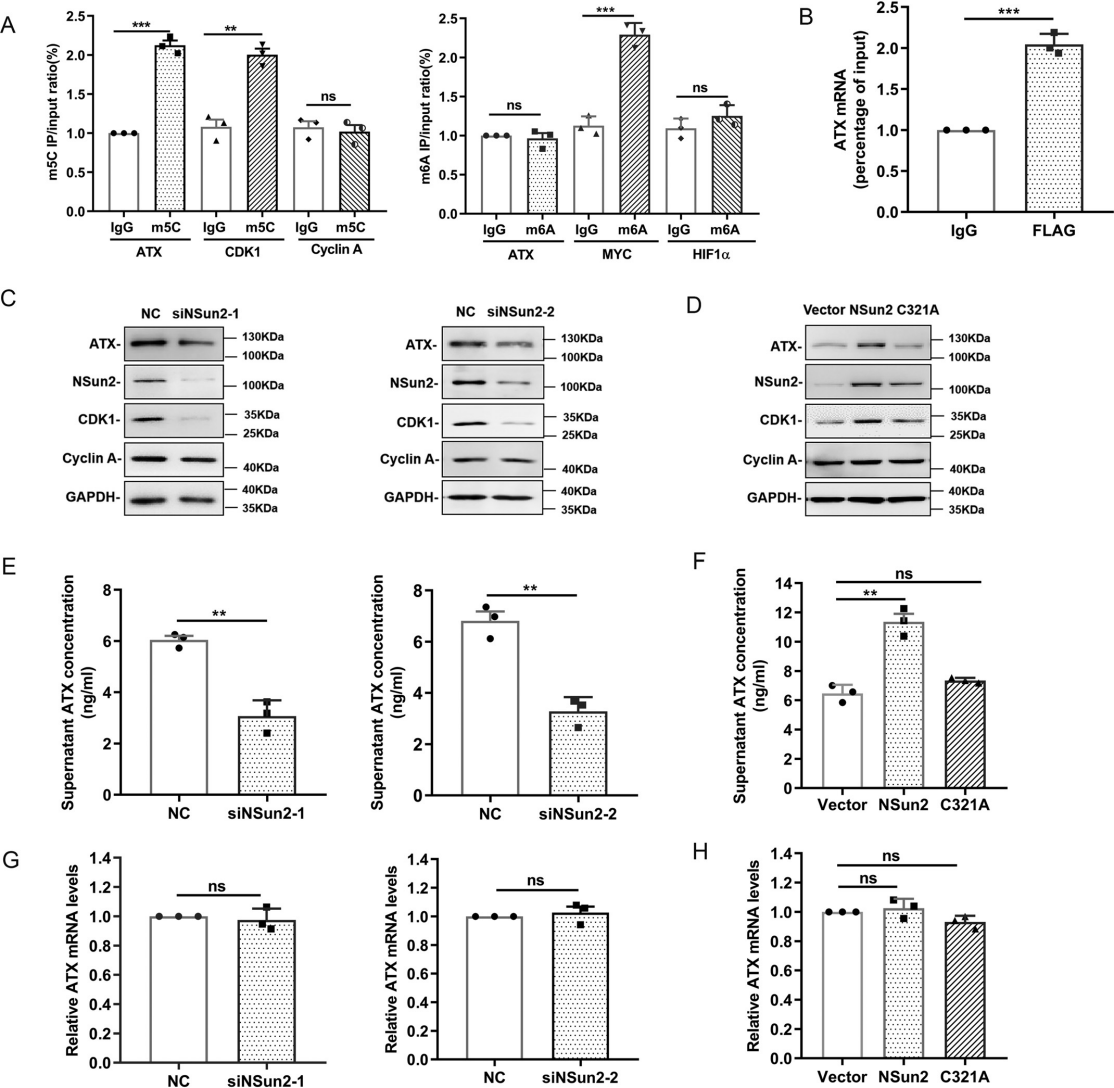
**NSun2 upregulates ATX protein expression.***A*, enrichment of endogenous m^5^C and m^6^A modifications in ATX mRNA in U87 cells. CDK1 and cyclin A served as the positive control and negative control of m^5^C IP, respectively, whereas MYC and HIF-1α were the counterparts of m^6^A IP. *B*, UV crosslink RNP-IP assays showed the association of ATX mRNA with FLAG-tagged NSun2 (C271A), a mutant tightly binding to target RNAs, in U87 cells. *C* and *D*, the effects of NSun2 knockdown or overexpression on ATX protein levels measured by Western blotting. U87 cells were transfected with negative control (*NC*) siRNA or two different NSun2 siRNAs, respectively (*C*). A vector expressing the WT NSun2 (pcDNA3.1-NSun2) or the RNA MTase enzyme-dead mutant NSun2 (C321A) was transfected into U87 cells (*D*). CDK1 was the positive control, cyclin A was the negative control, and GAPDH was the loading control. *E* and *F*, ATX concentration in the NSun2-silenced or -overexpressed cell culture medium was assessed via a human ATX ELISA kit. *G* and *H*, ATX mRNA levels in the NSun2-silenced or -overexpressed cells were analyzed by RT-qPCR and normalized to GAPDH mRNA levels. All the data are presented as the mean ± S.E. of *n* = 3 independent experiments. *p* values were calculated by two-sided unpaired Student's *t* test; *ns*, not significant; **p < 0.01; ***p < 0.001.

N6-methyladenosine (m^6^A) and m^5^C are two major types of mRNA methylation. To test whether m^6^A or m^5^C modification exists in ATX mRNA, ribonucleoprotein-immunoprecipitation (RNP-IP) assays were performed by using anti-m^5^C or -m^6^A antibody. CDK1 and cyclin A were the positive control and negative control of m^5^C IP, respectively, whereas MYC and HIF-1α were the counterparts of m^6^A IP (8, 25). As shown in Fig. 1*A*, ATX mRNA could be effectively enriched by anti-m^5^C antibody but not by anti-m^6^A antibody, suggesting that m^5^C modification may occur in ATX mRNA. Because NSun2 is a main mRNA methyltransferase catalyzing m^5^C formation, we further tested whether NSun2 could interact with ATX mRNA. To this end, a vector expressing FLAG-tagged NSun2 (C271A), which could tightly bind to target RNAs, was transfected into U87 cells. It was found by immunoprecipitation assays using anti-FLAG antibody that FLAG-tagged NSun2 (C271A) could enrich ATX mRNA, suggesting that NSun2 may directly bind to ATX mRNA (Fig. 1*B*). We further tested the protein levels of ATX from the cells with silenced NSun2 or overexpressed NSun2. As shown in Fig. 1*C*, knockdown of NSun2 decreased the protein levels of ATX. On the other hand, ectopic expression of NSun2, but not that of the RNA methyltransferase enzyme-dead mutant NSun2 (C321A), elevated the protein levels of ATX (Fig. 1*D*). The effect of NSun2 knockdown or overexpression on ATX protein levels was further confirmed by ELISA (Fig. 1, *E* and *F*). However, neither knockdown nor overexpression of NSun2 could alter the mRNA levels of ATX (Fig. 1, *G* and *H*). Apart from U87 cells, the effects of NSun2 intervention on ATX expression were also confirmed in colon cancer cells Colo320 and prostate cancer cells DU145 (Fig. S1). Therefore, NSun2 may regulate ATX expression at the post-transcriptional level.

### NSun2 methylates the 3′UTR of ATX mRNA

**Figure 2:**
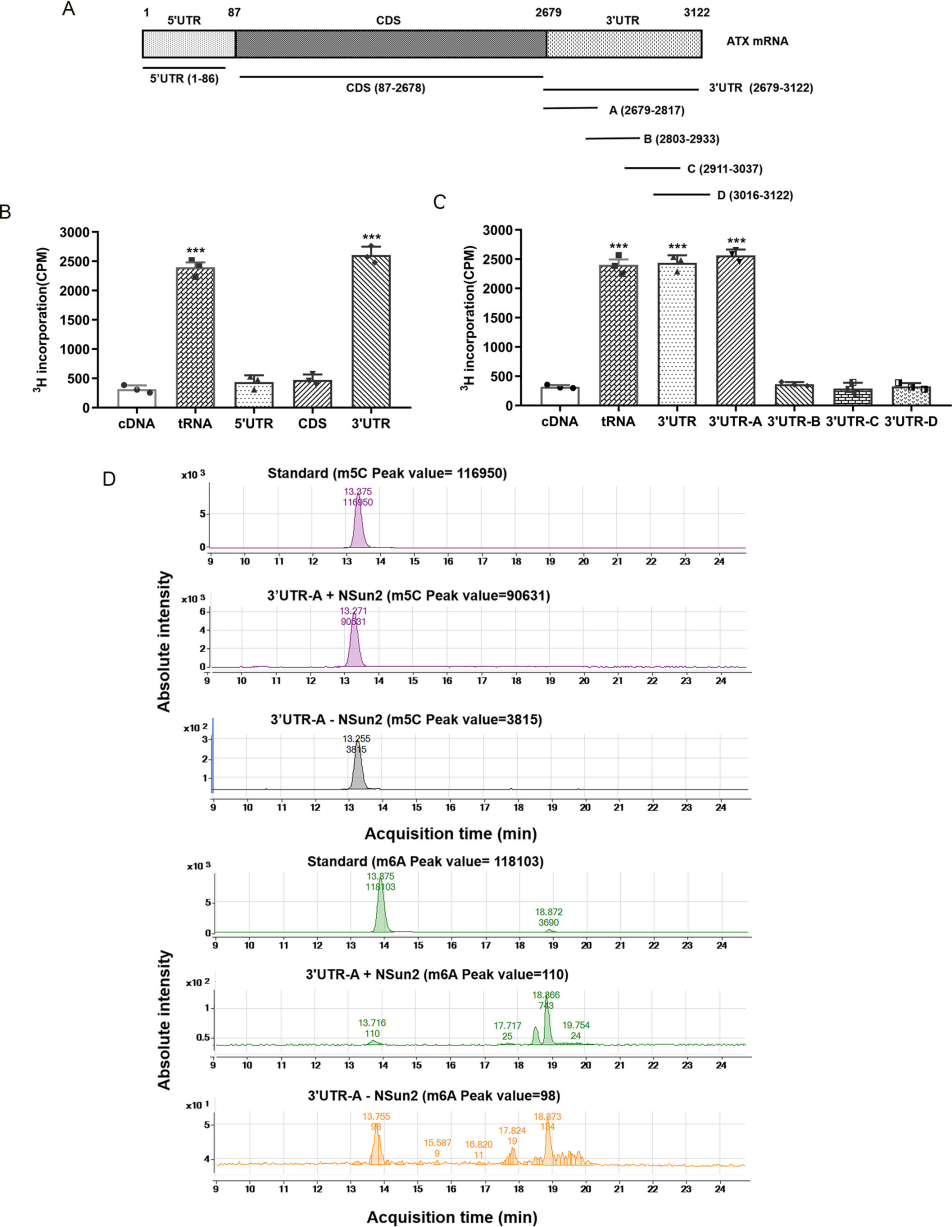
**NSun2 methylates ATX mRNA 3′UTR *in vitro*.***A*, schematic presentation of the fragments of ATX mRNA used in the *in vitro* methylation assays. *B*, incorporation of ^3^H-labeled SAM into ATX mRNA 5′UTR, CDS, and 3′UTR fragments. *C*, incorporation of ^3^H-labeled SAM into ATX mRNA full-length 3′UTR and 3′UTR-A, -B, -C, and -D fragments. ATX cDNA and bacterial tRNA were used as the negative and positive control, respectively. *D*, ATX mRNA 3′UTR-A fragments methylated *in vitro* by NSun2 or unmethylated were treated with nuclease P1 and alkaline phosphatase and then subjected to HPLC-MS assays. The presence of m^5^C and m^6^A was assessed. All the data are presented as the mean ± S.E. of the results of three independent experiments. *p* values were calculated by two-sided unpaired Student's *t* test; *ns*, not significant; ***p < 0.001.

To test whether NSun2 was capable of catalyzing ATX mRNA methylation, we used ^3^H-labeled SAM as methyl donor and performed *in vitro* methylation assays. The *in vitro* transcribed ATX mRNA fragments depicted in Fig. 2*A* were incubated with His-NSun2 protein and ^3^H-labeled SAM. ATX cDNA served as the negative control, whereas bacterial tRNA molecules were used as the positive control. As shown in Fig. 2, *B* and *C*, the methylation levels of ATX 3′UTR and 3′UTR-A were significantly higher than those of 5′UTR, coding sequence (CDS), 3′UTR-B, 3′UTR-C, and 3′UTR-D fragments. Furthermore, methylated and unmethylated 3′UTR-A fragments were subjected to HPLC-MS assays to determine the formation of m^5^C in ATX 3′UTR-A. As shown in Fig. 2*D*, m^5^C, but not m^6^A modification, was detected in the methylated ATX mRNA 3′UTR-A. These results indicate that ATX mRNA can be methylated by NSun2 *in vitro* and that the m^5^C methylation site is located in the ATX mRNA 3′UTR-A.

### NSun2 methylates ATX mRNA 3′UTR at C2756

**Figure 3:**
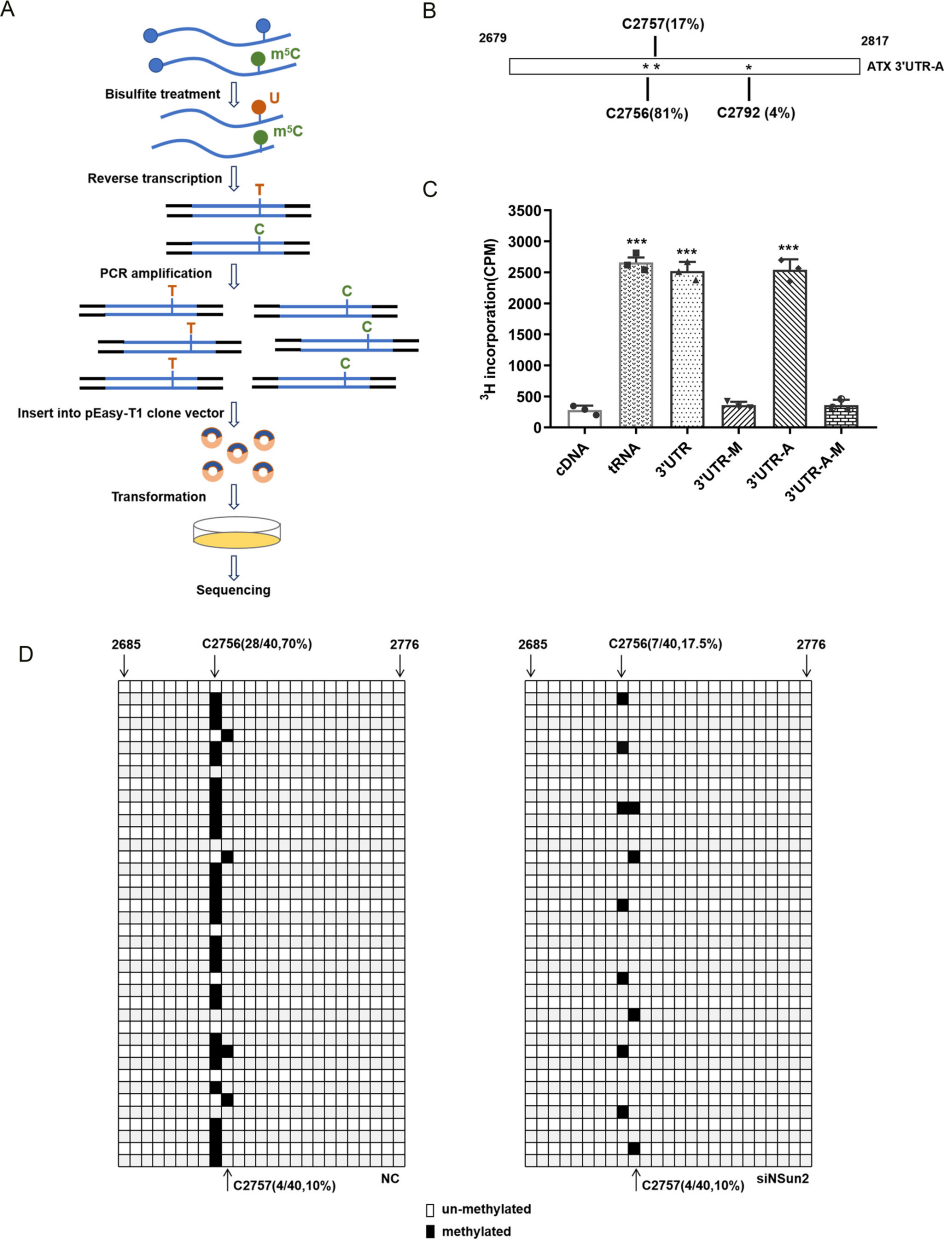
**NSun2 methylates ATX mRNA mainly at C2756.***A*, brief pattern diagram of bisulfite RNA-Seq method. *B*, *in vitro* transcribed ATX mRNA 3′UTR-A fragment was methylated by NSun2, and then bisulfite RNA-Seq analysis was conducted to identify the methylation site. The percentages of different m^5^C methylation sites in ATX mRNA 3′UTR-A were indicated. *C*, ATX mRNA 3′UTR, 3′UTR-A, 3′UTR-M (cytosine 2756 mutated to thymidine in 3′UTR), and 3′UTR-A-M (cytosine 2756 mutated to thymidine in 3′UTR-A) fragments were used to perform the *in vitro* methylation analysis. The incorporation of ^3^H-labeled SAM into each fragment was measured by liquid scintillation counting. ATX cDNA and bacterial tRNA served as the negative control and positive control, respectively. *D*, U87 cells were transfected with NC siRNA or NSun2 siRNA, and total RNA was isolated at 48 h after transfection. RNA was subjected to bisulfite sequencing analysis, and the levels of C2756 and C2757 methylation in ATX mRNA were assessed. Open boxes at positions of C2756 and C2757 indicate the unmethylated cytosines, which were converted to uracil and read as thymidine in ATX cDNA. Filled boxes at C2756 and C2757 indicate the methylated cytosines, which retained as cytosine in ATX cDNA. The numbers showed the residue positions in ATX mRNA with the first residue of 5′UTR as first. All the data are presented as the mean ± S.E. of the results of three independent experiments. *p* values were calculated by two-sided unpaired Student's *t* test; *ns*, not significant; ***p < 0.001.

To further identify the methylation site in the 3′UTR of ATX mRNA, the methylated ATX 3′UTR-A fragment was subjected to bisulfite RNA-Seq (Fig. 3*A*). As shown in Fig. 3*B*, three m^5^C sites were identified (C2756, 81%; C2757, 17%; and C2792, 4%), and C2756 was identified as the site with the highest methylation efficiency. By using *in vitro* methylation assays, mutation of C2756 in 3′UTR and 3′UTR-A fragments (3′UTR-M and 3′UTR-A-M with the C2756T mutation) greatly reduced their methylation rates (Fig. 3*C*). Furthermore, as shown in Fig. 3*D*, by bisulfite sequencing analysis, U87 cells silenced with NSun2 exhibited a much lower methylation rate at C2756 (17.5%) compared with that observed in control cells (70%). Although C2757 was mildly methylated by NSun2 *in vitro*, knockdown of NSun2 had no significant effect on the methylation of C2757 in cells (Fig. 3*D*). Therefore, C2756 in ATX 3′UTR was identified as the major methylation site by NSun2.

### Methylation by NSun2 enhances the translation of ATX mRNA

**Figure 4:**
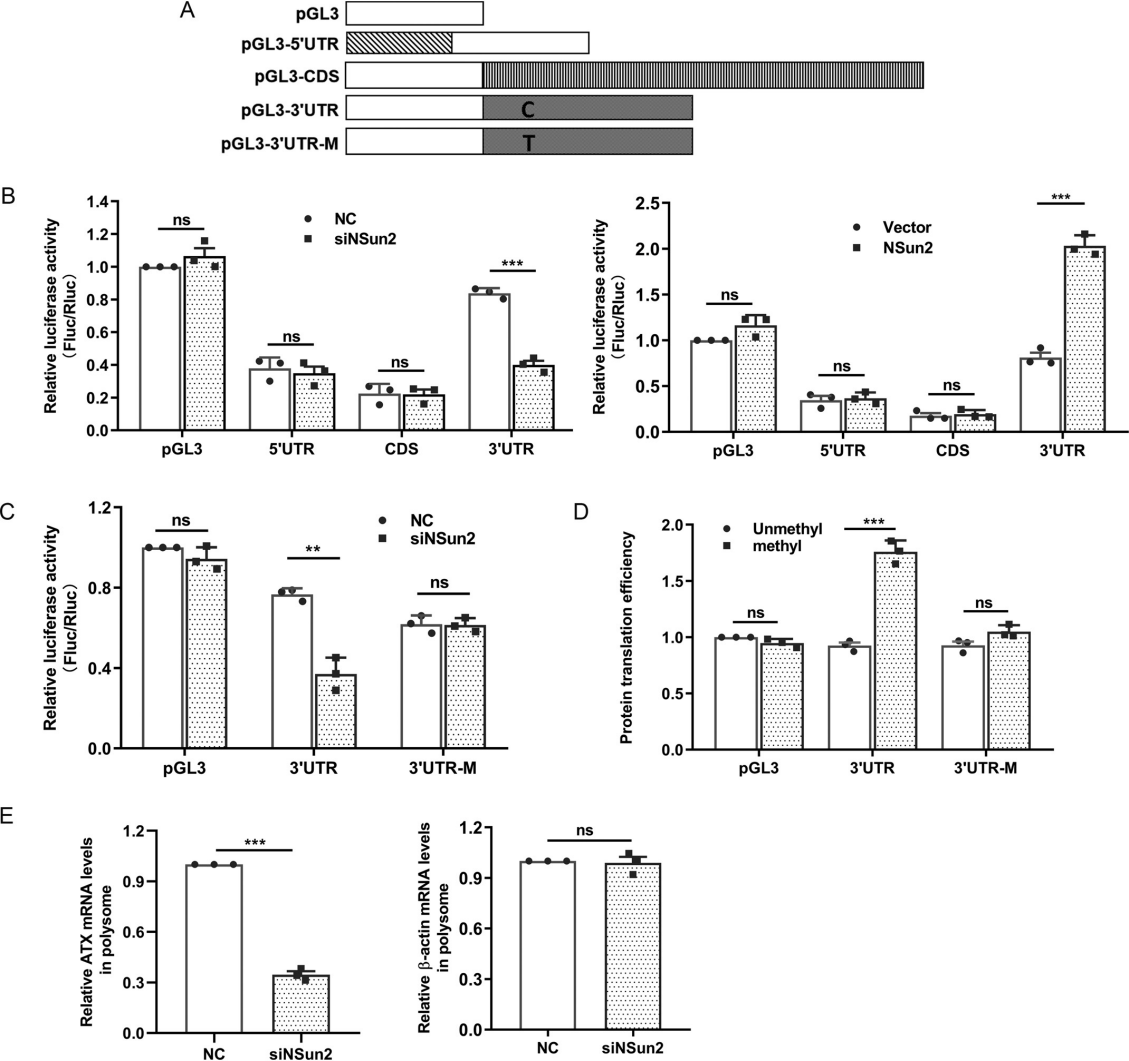
**Methylation of ATX mRNA by NSun2 enhances translation.***A*, schematic representation of the pGL3-derived reporter vectors used for reporter gene assays. *B*, HEK293T cells were transfected with pGL3 luciferase reporter vector fused to the indicated ATX fragment for 24 h, and then the cells were transfected with NSun2 siRNA (*left*) or a plasmid expressing NSun2 (pcDNA3.1-NSun2) (*right*). At 48 h later, each cell lysate was collected, and the luciferase activity was detected. Firefly luciferase activity (*Fluc*) was measured and normalized to Renilla luciferase activity (*Rluc*). *C*, the pGL3 luciferase reporter vector fused to ATX 3′UTR or 3′UTR-M (cytosine 2756 mutated to thymidine in 3′UTR) was transfected into HEK293T cells, which were then transfected with NC siRNA or NSun2 siRNA. Luciferase activity in each cell lysate was detected at 48 h after siRNA transfection. *D*, the luciferase transcripts from pGL3, pGL3-3′UTR, and pGL3-3′UTR-M were methylated by NSun2 or unmethylated (same reaction system but without adding NSun2) *in vitro*. The methylated and unmethylated reporter transcripts were used for *in vitro* translation assays. Luciferase activity was measured to reflect the translation efficiency. *E*, U87 cells treated with NC or NSun2 siRNA for 48 h were used to isolate the polysomal and nonpolysomal fractions. RNA was extracted from the polysomal fraction, and RT-qPCR analysis was performed to measure the content of ATX mRNA and β-actin mRNA in polysomal fraction. All the data are presented as the mean ± S.E. of *n* = 3 independent experiments. *p* values were calculated by two-sided unpaired Student's *t* test; *ns*, not significant; **p < 0.01; ***p < 0.001.

Next, we tested the role of NSun2-mediated ATX mRNA 3′UTR methylation in the regulation of protein expression. To this end, a series of pGL3-derived reporter vectors bearing different fragments of ATX cDNA were constructed (Fig. 4*A*). By using reporter gene assays, we found that the luciferase activity of pGL3-derived reporter bearing ATX 3′UTR, but not that bearing ATX 5′UTR, CDS, or 3′UTR-M (3′UTR with the C2756T mutation), was reduced in cells with silenced NSun2 and increased in cells with overexpressed NSun2 (Fig. 4, *B* and *C*). However, the luciferase mRNA levels were not changed (Fig. S2). These data suggest that methylation by NSun2 in ATX 3′UTR may have a positive effect on the expression of ATX at the post-transcriptional level.

Because intervention of NSun2 expression in cells did not alter the levels of ATX mRNA (Fig. 1, *G* and *H* and Fig. S1, B and D), we performed *in vitro* translation assays to confirm whether NSun2-mediated ATX mRNA 3′UTR methylation could regulate protein expression at the translational level. The transcripts transcribed from pGL3, pGL3-3′UTR, and pGL3-3′UTR-M (C2756T) were methylated by NSun2 *in vitro* or kept unmethylated. These transcripts then were used for *in vitro* translation assays. The luciferase activity was quantified as the readout of translation efficiency. As shown in Fig. 4*D*, the luciferase activity observed from the methylated pGL3-3′UTR transcripts was significantly higher than that from the unmethylated pGL3-3′UTR transcripts. Mutation of C2756 in 3′UTR abolished the effect of methylation in promoting the reporter activity (Fig. 4*D*). Furthermore, knockdown of NSun2 reduced the recruitment of ATX mRNA to polysomes (Fig. 4*E*). Together, by methylating ATX mRNA at the 3′UTR, NSun2 may enhance the expression of ATX at the translational level.

### Methylation by NSun2 promotes the nuclear export of ATX mRNA

**Figure 5:**
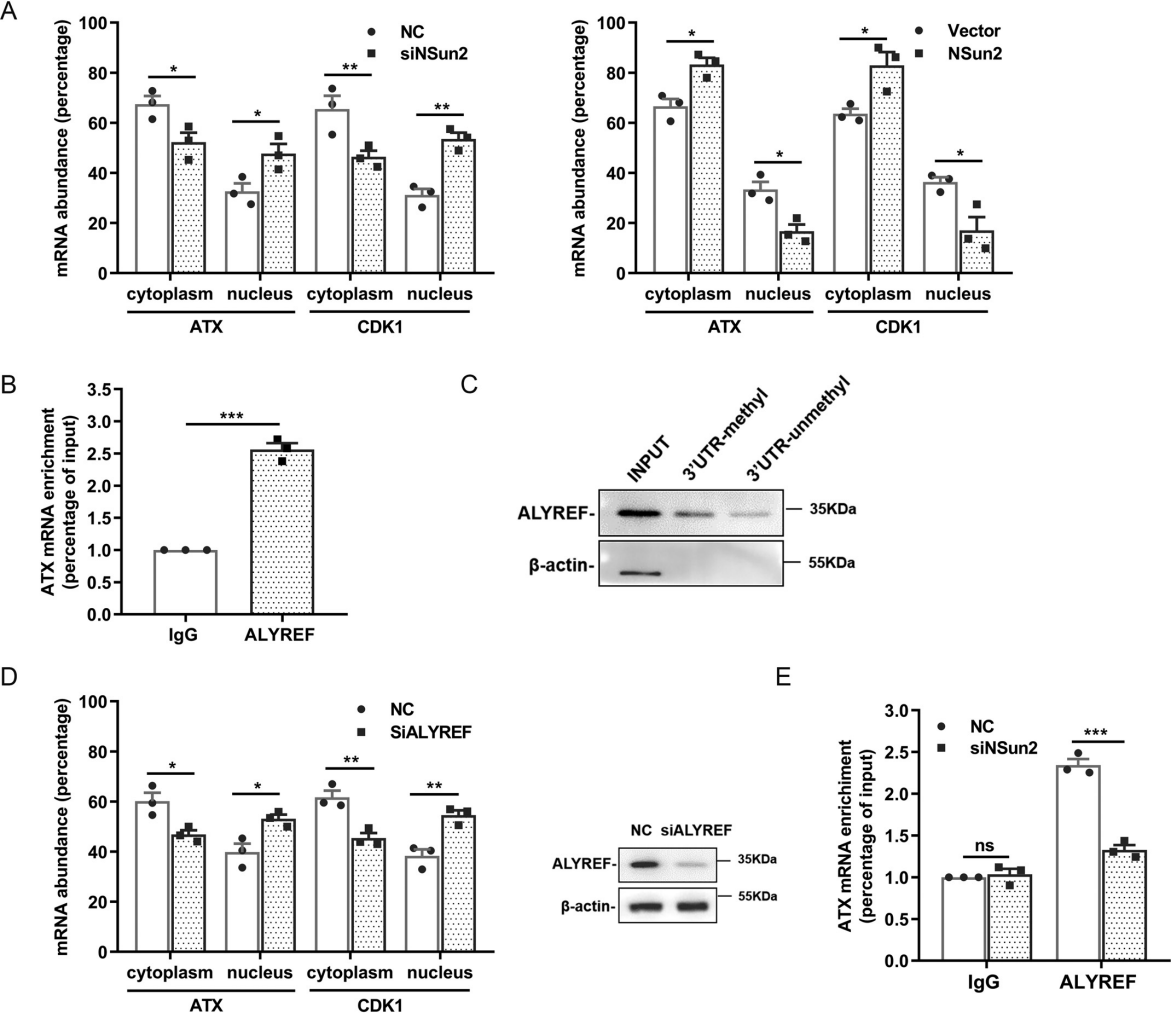
**Methylation by NSun2 promotes the nuclear export of ATX mRNA in an ALYREF-dependent manner.***A*, the percentage content of cytoplasmic and nuclear ATX mRNA was analyzed by RT-qPCR in U87 cells, which were transfected with NSun2 siRNA (*left*) or a plasmid expressing NSun2 (pcDNA3.1-NSun2) (*right*). CDK1 mRNA served as a positive control. *B*, the interaction between the endogenous ATX mRNA and ALYREF was analyzed by UV crosslink RNP-IP assays. *C*, demonstration of endogenous ALYREF in U87 cells pulled down by biotin-labeled ATX mRNA 3′UTR fragments methylated or unmethylated. *D*, the percentage content of cytoplasmic and nuclear ATX mRNA in ALYREF-silenced U87 cells was analyzed by RT-qPCR, with CDK1 mRNA as a positive control (*left*). The knockdown efficiency of ALYREF by siRNA was confirmed by Western blotting (*right*). *E*, the interaction between the endogenous ATX mRNA and ALYREF in NSun2-silenced U87 cells was analyzed by UV crosslink RNP-IP assays. Nonspecific IgG was used as the negative control. All the data are presented as the mean ± S.E. of *n* = 3 independent experiments. *p* values were calculated by two-sided unpaired Student's *t* test; *ns*, not significant; *p < 0.05; **p < 0.01; ***p < 0.001.

It has been reported that m^5^C formation promotes the nuclear export of mRNA (26). To validate whether NSun2-mediated mRNA methylation promotes the nuclear export of ATX mRNA, the relative abundance of cytoplasmic and nuclear ATX mRNA in cells with silenced or overexpressed NSun2 was tested. As shown in Fig. 5*A*, knockdown of NSun2 in U87 cells significantly decreased the levels of ATX mRNA in cytoplasm and increased the levels of ATX mRNA in nucleus. On the other hand, overexpression of NSun2 increased the presence of ATX mRNA in cytoplasm but reduced its presence in nucleus. Similar results were obtained in Colo320 and DU145 cells (Fig. S3).

Because RNA binding protein ALYREF is necessary for the nuclear export of m^5^C-modified mRNAs (26), the role of ALYREF in NSun2-regulated nuclear export of ATX mRNA was further examined. Interestingly, ALYREF could interact with ATX mRNA in cells (Fig. 5*B*) and was prone to associate with methylated ATX 3′UTR fragment (Fig. 5*C*). The cytoplasmic ATX mRNA levels were decreased in U87 cells with silenced ALYREF (Fig. 5*D*). Knockdown of NSun2 decreased the association of ATX mRNA with ALYREF (Fig. 5*E*). These data indicate that NSun2-mediated m^5^C methylation promotes the export of ATX mRNA from nucleus to cytoplasm in an ALYREF-dependent manner.

### NSun2-ATX-LPA axis impacts cell migration

**Figure 6:**
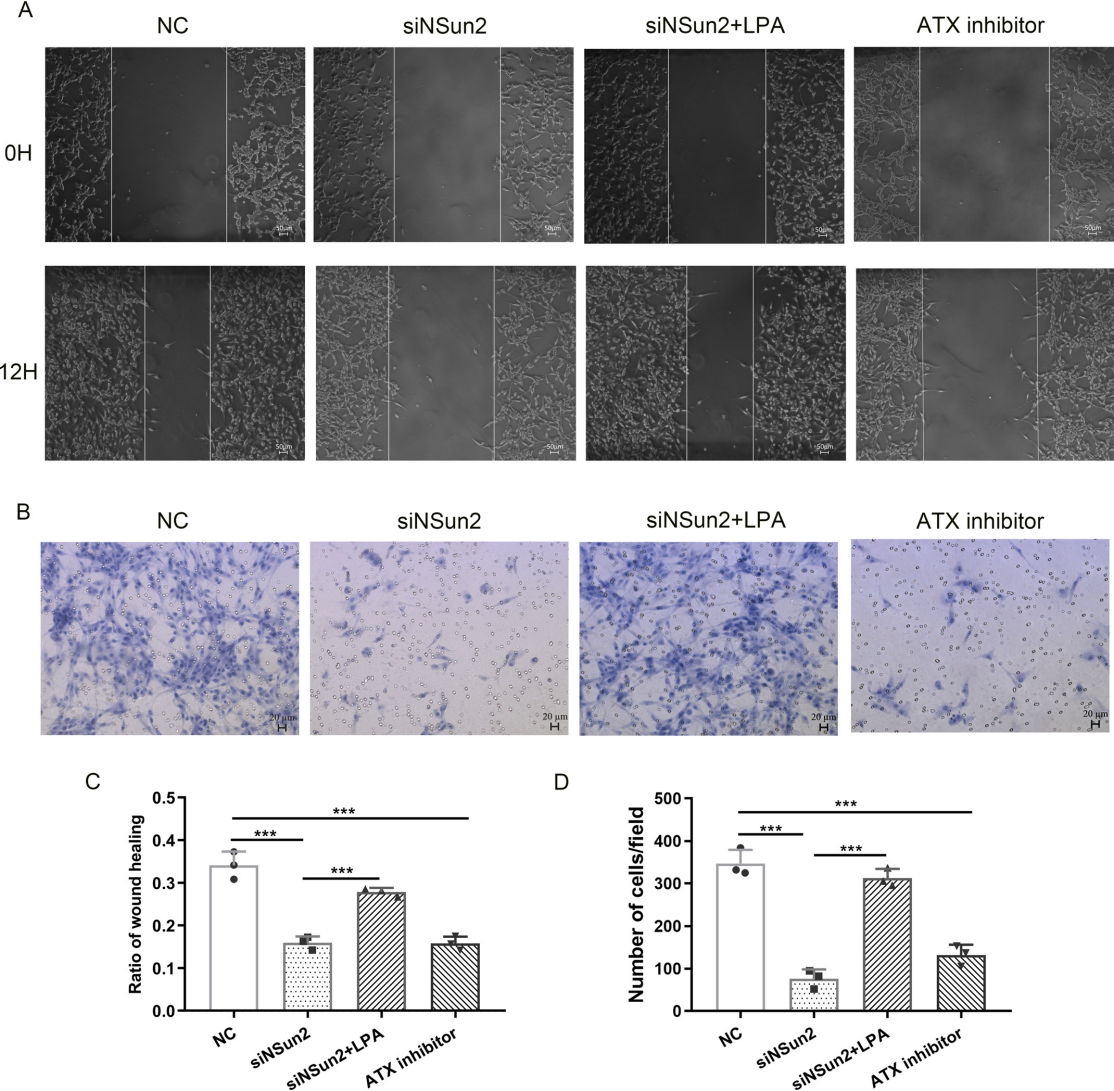
**The effects of NSun2 knockdown on U87 cell migration.***A* and *C*, scratch wound-healing assays. U87 cells were treated with the indicated negative control siRNA (*NC*), NSun2 siRNA, NSun2 siRNA with LPA (2 µm), or ATX inhibitor S32826 (2 µm). Images of the cells migrating into the wound were captured at 0 and 12 h with an inverted microscope (*A*). The relative migration rate was calculated via dividing the change of the gap distance between the scratch edges by the initial distance (*C*). *B* and *D*, transwell assays. U87 cells were treated with NC siRNA, NSun2 siRNA, NSun2 siRNA with LPA (2 µm), or ATX inhibitor S32826 (2 µm) as indicated, and then subjected to the transwell assays. Images of cells on the lower surface of upper chamber were taken 24 h after the cells were seeded into the upper chamber (*B*). The relative migration was calculated by counting the number of cells on the lower surface of the upper chamber, and data were obtained from three randomly chosen fields (*D*). All the data are presented as the mean ± S.E. of *n* = 3 independent experiments. *p* values were calculated by two-sided unpaired Student's *t* test; *ns*, not significant; ***p < 0.001.

NSun2 is highly expressed in various cancer cells. Our results suggest that m^5^C methylation of ATX mRNA by NSun2 increases the expression of ATX, the key enzyme for extracellular LPA production. In addition, it has been reported that the ATX-LPA axis can promote cell migration (15, 16). To illustrate the effects of the NSun2-ATX regulatory process on cell migration, wound-healing and transwell assays were performed. Fig. 6 showed that the migration of U87 cells was inhibited when NSun2 was silenced, in accordance with the findings that the ATX inhibitor S32826, a potent dose-dependent inhibitor that is nontoxic to cells at less than 10 μm (27, 28, 29), inhibited U87 cell migration at 2 μm. In addition, administration of LPA in culture medium rescued the effect of NSun2 knockdown in inhibiting cell migration.

**Figure 7:**
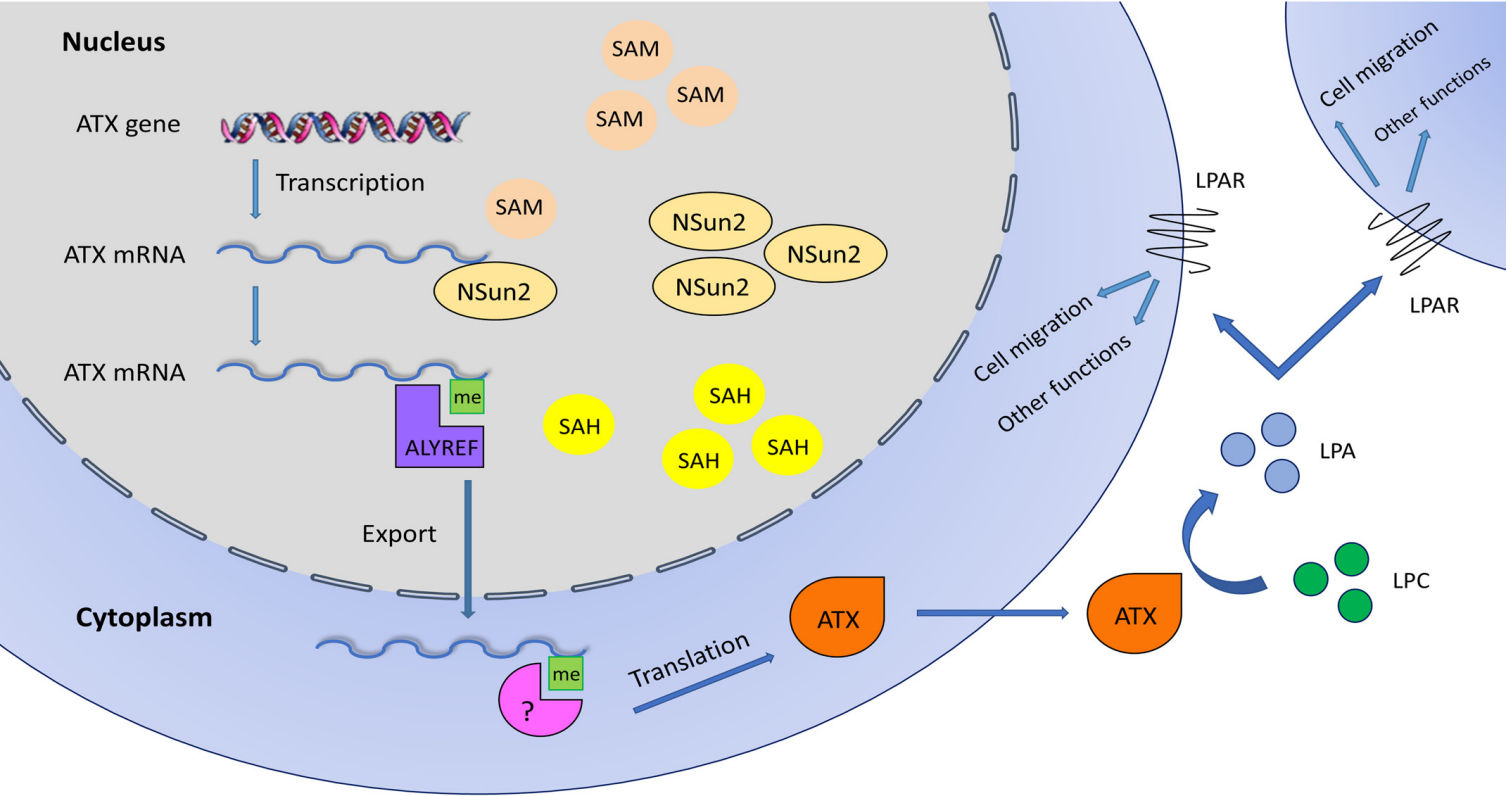
**Regulation of ATX-LPA axis by NSun2**. NSun2 methylates ATX mRNA at 3′UTR and promotes the export of ATX mRNA from nucleus to cytoplasm in an ALYREF-dependent manner. The m^5^C methylation by NSun2 also enhances ATX mRNA translation. The m^5^C reader protein of methylated ATX mRNA in cytoplasm remains to be identified. The increase of ATX protein expression leads to the elevation of extracellular levels of LPA, which promotes cell migration through LPA receptors on cell surface. *LPAR*, LPA receptor.

In conclusion, the current study indicates that methylation of ATX mRNA 3′UTR by NSun2 promotes ATX mRNA export from nucleus to cytoplasm in an ALYREF-dependent manner and enhances ATX expression at the translational level. The NSun2-ATX regulatory process impacts cell migration (Fig. 7).

## Discussion

By methylating different RNAs, NSun2 is able to regulate cell proliferation, differentiation, and senescence. Studies have reported that the protein level of NSun2 elevates in several kinds of cancers, such as colorectal cancers, glioblastoma, and so on (30). However, the role of NSUN2 in cancers and the mechanisms underlying are largely unknown. ATX is a secreted glycoprotein that catalyzes the conversion of lysophosphatidylcholine into LPA. The ATX-LPA pathway plays a vital role in cancer development, especially in cancer cell migration. In the present study, we demonstrate that NSun2 is able to methylate ATX mRNA in 3′UTR to enhance ATX expression at the post-transcriptional level. Knockdown of NSun2 led to the suppression of cell migration, which could be recovered by the addition of LPA. These data indicate that NSun2 participates in cell migration through regulating the ATX-LPA axis.

ALYREF can specifically recognize m^5^C on mRNAs and facilitate the nuclear export of target mRNAs as an adaptor (26). Our findings indicate that ALYREF is prone to associate with the methylated ATX 3′UTR fragment, in turn enhancing ATX translation through promoting the nuclear export of ATX mRNA. Knockdown of NSun2 and ALYREF together exhibited a stronger effect than knockdown NSun2 along in reducing the presence of ATX mRNA in polysomes (Fig. S4), suggesting that ALYREF may contribute to the assembly of ATX mRNA in polysomes. However, whether ALYREF is directly involved in the promotion of ATX protein translation in cytoplasm is still unknown and remains to be further explored.

In addition to ALYREF, YBX1 is an m^5^C reader protein of methylated mRNA that has been reported (13). We explored the effects of YBX1 to ATX expression. However, we did not detect any notably difference in ATX protein or mRNA levels after knocking down YBX1 compared with control ones (Fig. S5, A–C). RNP-IP assays demonstrated that YBX1 may not interact with ATX mRNA (Fig. S5D). These results indicate that YBX1 is not the reader protein of methylated ATX mRNA and that other m^5^C reader proteins of methylated ATX mRNA may exist.

NSun2 is highly expressed in neuroepithelial progenitors in the neural tube (31). Loss of function of the NSun2 gene impairs brain development during early embryo formation stage and causes microcephaly in mouse and human (32, 33). Interestingly, ATX is also highly expressed in the developing mouse cortex, and ATX knockdown inhibits the neuronal progenitor adhesion and positioning (34). NSun2 and ATX coexist in the neuronal progenitors, and both participate in the development of the brain. Therefore, the NSun2-ATX regulatory process may be also involved in the aforementioned processes. In addition, NSun2 may take part in other biological processes through modulating the ATX-LPA axis, which remains to be explored in the future.

## Experimental procedures

### Antibodies

The NSun2 antibody, cyclin A antibody, β-actin antibody, and GAPDH antibody were all purchased from Santa Cruz Biotechnology. The CDK1 antibody and FLAG antibody were purchased from Cell Signaling Technology. The ALYREF antibody, YBX1 antibody, IgG antibody, anti-m^5^C antibody, and anti-m^6^A antibody were purchased from Abcam. The ATX primary antibody was generated as previously described (35).

### Cell culture and transfection

U87, DU145, and HEK293T cells were cultured in DMEM (Macgene, Beijing, China). Colo320 cells were maintained in RPMI 1640 medium (Macgene, Beijing, China). The medium was supplemented with 10% FBS (Biowest, Riverside, MO), 100 units/ml penicillin, and 0.1 mg/ml streptomycin (Thermo Fisher Scientific). All cells were cultured at 37 °C in 5% CO_2_. The plasmids and siRNAs were transfected using Lipofectamine 2000 (Thermo Fisher Scientific) following the manufacturer's instructions. The siRNAs used were 5′-GAGAUCCUCUUCUAUGAUCTT-3′ for siNSun2-1; 5′-GGAGAACAAGCUGUUCGAGTT-3′ for siNSun2-2; 5′-GAGGUGGCAUGACUAGAAATT-3′ for siALYREF; and 5′-GGAACGGAUAUGGUUUCAUTT-3′ for siYBX1. Two NSun2 siRNAs were used in Fig. 1, and siNSun2-1 was used in following other experiments. To detect secreted ATX protein, the cells were cultured in serum-free medium for 24 h, and then the supernatant of medium was collected to detect ATX content using a human ATX ELISA kit or by Western blotting analysis.

### Western blotting analysis and ELISA

Whole-cell lysates were prepared in radioimmune precipitation assay buffer for 30 min. After centrifugation at 4 °C, the supernatants were quantified by bicinchoninic acid assays (Pierce^TM^ BCA Protein Assays Kit, Thermo Fisher Scientific). Protein samples were subjected to SDS-PAGE and analyzed with different antibody. For the detection of ATX in the culture medium by Western blotting, the cell culture medium was concentrated (20-fold) using an Amicon Ultra 30000 (Merck KGaA, Darmstadt, Germany). Protein quantification was conducted, and equal protein amounts were loaded for each sample. Human ATX ELISA kit was obtained from R&D Systems (Bio-Techne, Minneapolis, MN), and the provided instructions were followed.

### Total RNA isolation, nucleocytoplasmic fractionation of RNA, and RT-qPCR

Total cellular RNA was extracted from cells with TRIzol reagent (Merck KGaA, Darmstadt, Germany). RNA (1 μg) was reverse-transcribed by a reverse-transcription system (Promega) and then analyzed by quantitative real-time PCR assays. Nucleocytoplasmic fractionation of RNA was performed using the Ambion PARIS Protein and RNA Isolation System (Thermo Fisher Scientific) according to the manufacturer's instructions. The reverse-transcription products of nuclear and cytoplasmic RNA fractions were analyzed by PCR using 45S pre-rRNA and RPS14 mRNA as the nuclear and cytoplasmic RNA markers, respectively. The samples were further amplified by quantitative real-time PCR and analyzed. The primer pairs used for qPCR were 5′-TATGCTTCGGAAAGAAATGGAG-3′ and 5′-ATGTTCAATGTCACGCACCCT-3′ for ATX mRNA; 5′-GGTCAACCATGATGCCTCCA-3′ and 5′-GCGTCCCAGTCTGTAAACCA-3′ for NSun2 mRNA; 5′-CTGGGCTACACTGAGCACC-3′ and 5′-AAGTGGTCGTTGAGGGCAATG-3′ for GAPDH mRNA; 5′-CTGGGGTCAGCTCGTTACTC-3′ and 5′-TCCACTTCTGGCCACACTTC-3′ for CDK1 mRNA; 5′-CCGCGCTCTACCTTACCTAC-3′ and 5′-GAGCGACCAAAGGAACCATA-3′ for 45S pre-rRNA; 5′-GGCAGACCGAGATGAATCCTC-3′ and 5′-CAGGTCCAGGGGTCTTGGTCC-3′ for RPS14 mRNA; 5′-GGGCATGGGTCAGAAGGATT-3′ and 5′-TCGATGGGGTACTTCAGGGT-3′ for β-actin mRNA; 5′-GAGAAGTGATGGAGGGTGCT-3′ and 5′-TTAGGGTTTTCTGGGCGTCT-3′ for YBX1 mRNA; 5′-ATGAGACCCTGCATTTGGCT-3′ and 5′-CCCGTGACTGTGTAGAGTGC-3′ for cyclin A mRNA; 5′-GCATACATCCTGTCCGTCCA-3′ and 5′-GTCGTTTCCGCAACAAGTCC-3′ for MYC mRNA; and 5′-CGCAAGTCCTCAAAGCACAG-3′ and 5′-TCTGTTTGGTGAGGCTGTCC-3′ for HIF-1α mRNA. Each qPCR experiment was repeated at least three times with three replicate samples.

### Luciferase reporter construction and luciferase activity assays

To construct the luciferase reporter plasmids, the ATX 5′UTR, CDS, and 3′UTR, and the 3′UTR-A, -B, -C, and -D fragment primers, were used as previously described (32). The pGL3-ATX-3′UTR-M was obtained from the pGL3-ATX-3′UTR with the following site-mutation primers: 5′-TATTTATTAATTTGAAATCAGGACATTAAAAATGTT-3′ and 5′-ATTTTTAATGTCCTGATTTCAAATTAATAAATACAA-3′. HEK293T cells (1 × 10^5^) were plated in 24-well plates before being transfected with a mixture of luciferase reporter plasmid and Renilla luciferase plasmid, then 24 h later were transfected with negative control (NC) and NSun2 siRNA or empty vector and NSun2-expressing plasmid, respectively. The cells were collected at 48 h after transfection, and luciferase activity was detected by a Dual-luciferase Reporter Assays System Kit (Promega). Reporter gene activity was determined by normalization of firefly luciferase activity to Renilla luciferase activity. Firefly luciferase mRNA levels were tested by RT-qPCR with Renilla luciferase mRNA as control. Firefly luciferase primers: 5′-CACTCTGGCGACATTGCCTA-3′ and 5′-GCTGCAGCAGGATAGACTCC-3′. Renilla luciferase primers: 5′-GAGAAGGGCGAGGTTAGACG-3′ and 5′-TGGAAAAGAACCCAGGGTCG-3′.

### UV crosslink RNP-IP assays

For crosslink RNP-IP assays, antibody was incubated with protein G-Sepharose beads overnight in 4 °C with rotation. The cells were exposed to UVC (400 mJ/cm^2^), and the cellular extracts were incubated with the Ab-beads prepared before by rotating for 2 h in 4 °C for immunoprecipitation. After incubation, the beads were spun at 7000 × *g* for 5 min to discard the supernatant and then washed. RNA isolation and qPCR were performed as described above. The primers used in RNP-IP showed in the section of total RNA isolation, nucleocytoplasmic fractionation of RNA, and RT-qPCR.

### Preparation of in vitro transcription fragments

U87 cDNA was used as the template for PCR amplification of different fragments of ATX mRNA. The T7 promoter sequence CCAAGCTTCTAATACGACTCACTATAGGGAGA (T7) was included in all 5′ primers. To prepare templates for ATX 5′UTR (positions 1 to 86), CDS (positions 87 to 2678), 3′UTR (positions 2679 to 3122), and 3′UTR-A (positions 2679 to 2817), -B (positions 2803 to 2933), -C (positions 2911 to 3037), and -D (positions 3016 to 3122), the following primer pairs were used: 5′-(T7)AATAGACTAAACCCAGAGCCTC-3′ and 5′-GTCGAGGATTCTTGGAAAGC-3′ for 5′UTR; 5′-(T7)ATGGCAAGGAGGAGCTCGTTC-3′ and 5′-TTAAATCTCGCTCTCATATGTATG-3′ for CDS; 5′-(T7)CTTTCTGAGCATCTGCAGTAC-3′ and 5′-AAACCAGAAAAAACTGAATGTGTG-3′ for 3′UTR; 5′-(T7)CTTTCTGAGCATCTGCAGTAC-3′ and 5′-ATTCAGGCATAATATGTCAGATTTG-3′ for 3′UTR-A; 5′-(T7)ATATTATGCCTGAATGACTCCAC-3′ and 5′-AATCTGCAGCACCATTTAGAAGC-3′ for 3′UTR-B; 5′-(T7)GCTTCTAAATGGTGCTGCAG-3′ and 5′-CAGCAAATAAAGGCAACTTTAC-3′ for 3′UTR-C; and 5′-(T7)GTAAAGTTGCCTTTATTTGCTG-3′ and 5′-AAACCAGAAAAAACTGAATGTGTG-3′ for 3′UTR-D. pGL3-ATX-3′UTR-M was used as the template for PCR amplification of ATX-3′UTR-M fragment (the 3′UTR fragment with C2756T mutation) with the primers 5′-(T7)CTTTCTGAGCATCTGCAGTAC-3′ and 5′-AAACCAGAAAAAACTGAATGTGTG-3′, and ATX-3′UTR-A-M fragment (the 3′UTR-A fragment with C2756T mutation) with the primers 5′-(T7)CTTTCTGAGCATCTGCAGTAC-3′ and 5′-ATTCAGGCATAATATGTCAGATTTG-3′. The *in vitro* transcription was performed by following the manufacturer's instruction of TranscriptAid T7 High Yield Transcription kit (Thermo Fisher Scientific).

### RNA pulldown assays

For biotin pulldown assays, PCR-amplified DNAs were used for the *in vitro* transcription of RNA probes in the presence of biotin-UTP (Biotium, Fremont, CA). One microgram of purified biotinylated transcripts was incubated with 100 μg of whole-cell lysates for 30 min at room temperature. Complexes were isolated with paramagnetic streptavidin-conjugated Dynabeads (Thermo Fisher Scientific), and the pulldown samples were analyzed by Western blotting analysis.

### In vitro methylation

To perform the *in vitro* methylation assays with ^3^H-labeled SAM (Amersham Biosciences), various transcriptional fragments of ATX, including 5′UTR, CDS, 3′UTR, 3′UTR-A, 3′UTR-B, 3′UTR-C, 3′UTR-D, 3′UTR-A-M, and 3′UTR-M, were prepared and used as the RNA probes *in vitro* as described above under “Preparation of *in vitro* transcription fragments.” His-tagged NSun2 was expressed in *Escherichia coli* and purified. Reaction mixtures (50 μl) containing 0.2 nmol/liter His-NSun2, 0.01 nmol/L RNA, and 1 μCi of ^3^H-labeled SAM in a reaction buffer (5 mmol/L Tris-HCl, pH 7.5, 5 mmol/liter EDTA, 10% glycerol, 1.5 mmol/liter DTT, and 5 mmol/liter MgCl_2_) supplemented with inhibitors (1 μg/ml leupeptin, 1 μg/ml aprotinin, 0.5 mmol/liter phenylmethylsulfonyl fluoride, and 5 units/μl RNasin) were incubated for 30 min at 37 °C. *E. coli* tRNA (0.01 nmol/liter; Merck KGaA) and ATX cDNA (0.01 nmol/liter) were used as the positive and negative control, respectively. The unincorporated ^3^H SAM was removed by using QiaQuick Spin Columns (Qiagen), and the incorporated radioactivity was measured by liquid scintillation counting.

### HPLC-MS analysis

The ATX mRNA 3′UTR-A fragments were methylated *in vitro* by NSun2 by using nonisotopic SAM (Merck KGaA). The *In vitro* methylated ATX mRNA 3′UTR-A fragment (1 μg) was digested with nuclease P1 (Merck KGaA) and alkaline phosphatase (Promega). HPLC-MS was performed to analyze the formation of m^5^C or m^6^A at the Tsinghua University Mass Spectrometry Center (Beijing, China).

### Bisulfite RNA-Seq

RNA samples were dissolved in 10 μl of RNase-free water, and a 2-μg volume of the dissolved sample was mixed with 42.5 μl of 5 m sodium bisulfite and 17.5 μl of DNA protection buffer from EpiTect Bisulfite Kit (Qiagen). Then, the mixture was repeatedly incubated at 70 °C for 5 min and 60 °C for 60 min for 3–5 cycles. After desalting using Micro Bio-Spin P-6 Gel columns (Bio-Rad), the samples were desulfonated by 1 m Tris, pH 9.0 (1/1, V/V) at 37 °C for 1 h, followed by ethanol precipitation (36). The bisulfite-converted fragments dissolved in 10 μl of RNase-free water were reverse-transcribed with a Revert Aid First Strand cDNA Synthesis Kit (Thermo Fisher Scientific). The reverse-transcribed products (cDNA) were subjected to PCR, and then the PCR products were inserted into the pEasy-T1 Cloning Vector System (TransGen Biotech, Beijing, China). The plasmids purified from single clones were sequenced. Sequencing results were aligned with the corresponding mRNA sequence, and the cytosines retained were considered to be methylated; otherwise, unmethylated cytosines were converted to uracil and read as thymidine in cDNA.

### In vitro methylation site assays

*In vitro* methylation site assays were performed to identify the m^5^C modification sites in ATX mRNA 3′UTR-A fragment. Briefly, the DNA template of 3′UTR-A fragment (positions 2679 to 2817) was amplified by using the primers 5′-(T7)GAGAGCGAGATTTAACTTTC-3′ and 5′-TCTTAACCTTCCTACCATTCCATTCAGGCATAATATGTCAG-3′. This fragment (1 μg) was transcribed *in vitro* and methylated by NSun2 by using nonisotopic SAM (Merck KGaA), then subjected to the bisulfite RNA-Seq. During bisulfite RNA-Seq assay, the bisulfite-converted ATX mRNA 3′UTR-A fragments were reverse-transcribed by using the primer 5′-GTCGTATCCAGTGCAGGGTCCGAGGTATTCGCACTGGATACGACTCTTAACCTTCCTACCATTCC-3′, and then the products were amplified by PCR by using the primers 5′-GGGAGAGAGAGCGAGATTTAA-3′ and 5′-GCAGGGTCCGAGGTATTC-3′.

### In vivo methylation site assays

Total RNA (2 μg) from U87 cells was bisulfite-converted as described above under “Bisulfite RNA sequencing.” The converted ATX 3′UTR was reverse-transcribed by using the primer 5′-GCGTCTCAACTGGTGTCGTGGAGTCGGCAATTCAGTTGAGACGCCTAACATTTTT-3′. Then the reverse-transcribed products were amplified by PCR by using the primers 5′-CGGAGTATTTGTAGTATAGTTTTATTAATTGG-3′ and 5′-TGGTGTCGTGGAGTCGGC-3′. The PCR products were analyzed as described under “Bisulfite RNA sequencing.”

### In vitro translation assays

The luciferase (Luc) transcript was transcribed *in vitro* from the DNA fragment amplified from pGL3-control by using the primer pairs 5′-(T7)ATGGAAGACGCCAAAAACAT-3′ and 5′-TTTTTTTTTTTTTTTTTTTTTTTTTTTTTTACACGGCGATCTTTCCGCCCT-3′. The Luc-3ʹUTR and Luc-3ʹUTR-M transcripts were transcribed *in vitro* from the DNA fragments amplified from the pGL3-3ʹUTR and pGL3-3ʹUTR-M reporter vectors, respectively, by using primer pair 5′-(T7)ATGGAAGACGCCAAAAACAT-3′ and 5′-TTTTTTTTTTTTTTTTTTTTTTTTTTTTAAACCAGAAAAAACTGAATGTGTG-3′. Transcripts (0.01 nm) were either methylated by NSun2 or left untreated. The methylated and unmethylated transcripts were used for the *in vitro* translation assays with a rabbit reticulocyte lysate cell-free translation system (Promega). The translation efficiency was determined by measuring the activity of firefly luciferase.

### Preparation of the polysomal fractions

About 2 × 10^7^cells were prepared to incubate with 100 μg/ml cycloheximide for 15 min, and then total lysates were layered onto 30% sucrose in ice-cold buffer containing 20 mm HEPES, pH 7.4, 50 mm potassium acetate, 5 mm magnesium acetate, 1 mm DTT, 1 unit of RNasin/μl, 1 μg of leupeptin/ml, 1 μg of aprotinin/ml, and 0.5 mm phenylmethylsulfonyl fluoride. The lysates were centrifuged at 30,000 rpm for 2 h at 4 °C (Beckman Optima L-100), and RNA was isolated from the pellet (polysomal fraction) and analyzed by RT-qPCR.

### Transwell assays and scratch wound-healing assays

U87 cells were transfected with NSun2 siRNA and NC siRNA, respectively. For transwell assays, cell migration was measured based on the ability of the cells to migrate across a Transwell filter (8-μm pores, Costar, Cambridge, MA). After siRNA transfection for 48 h, U87 cells (4 × 10^4^) were suspended in 200 μl of serum-free DMEM and added to the upper chamber. The 10% FBS DMEM medium was added to the lower chamber. The ATX inhibitor S32826 (2 μm) or 18:1 LPA (2 μm) was added in the medium in the upper and lower chambers as indicated. After incubating for 24 h, the nonmigrated cells were scraped off from the filter using a cotton swab, and the cells that migrated to the lower side of the upper chamber were fixed with 4% paraformaldehyde and stained with hematoxylin. The cells per microscopic field (U87 cells, 20×) were imaged and counted in three randomly chosen fields. For the scratch wound-healing assays, 24 h after siRNA transfection, U87 cells were digested with trypsin enzyme and then seeded equally into six-well tissue culture plates and grown to almost total confluence at 24 h. The cells were starved for 8 h, and then artificial homogenous wounds were created on the monolayer with a sterile 10-μl tip. After scratching, the cells were incubated with serum-free medium in the absence or presence of ATX inhibitor S32826 (2 μm) or 18:1 LPA (2 μm) as indicated. Images of the cells migrating into the wound were captured at 0 and 12 h by an inverted microscope (10×), and all experiments were repeated at least three times.

## Statistical analysis

All data are shown as the mean ± S.E. of *n* = 3 independent experiments. GraphPad Prism software was used for data analysis. Statistical analysis was performed with two-sided unpaired Student's *t* test. A value of *p* < 0.05 was considered statistically significant.

## Data availability

All data are contained within this article and in the supporting information.
