# Supplementary material for: NSun2 promotes cell migration through methylating autotaxin mRNA
Source: J Biol Chem. 2021 Jan 13;295(52):18134–47. doi: 10.1074/jbc.RA119.012009 (PMC7939462; doi:10.1074/jbc.RA119.012009)
Supplement: Supplementary file 1 [file mmc1.pdf]

Supporting Information

NSun2 promotes cell migration through methylating autotaxin mRNA

Xin Xu[1], Yihua Zhang[1], Junjie Zhang[1, 2]*, and Xiaotian Zhang[1]*

[1]The Key Laboratory of Cell Proliferation and Regulation Biology, Ministry of Education, Institute of Cell Biology, College of Life Sciences, Beijing Normal University, Beijing 100875, China; [2]Academy of Plateau Science and Sustainability, People's Goverment of Qinghai Province & Beijing Normal University, Xining 810008, China.

File contents:

Figure S1-5

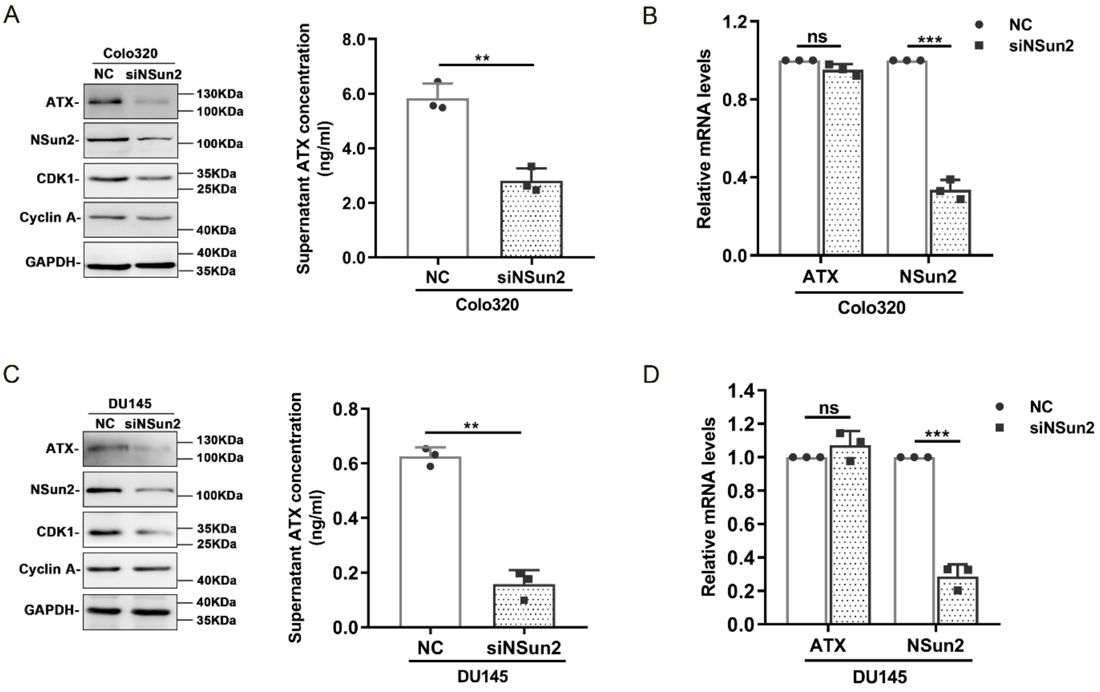

Figure S1. Effects of NSun2 on ATX expression in Colo320 and DU145 cells. Colo320 and DU145 cells were treated with NC or NSun2 siRNA as indicated. (A) and (C) ATX protein levels in cell culture medium and NSun2 protein levels in cells were tested by Western blot, with CDK1 as the positive control, Cyclin A as the negative control, and GAPDH as the loading control (left). ATX concentration in cell culture medium was further assessed via a human ATX ELISA kit (right). (B) and (D) ATX and NSun2 mRNA levels were detected in the indicated Colo320 cells (B) and DU145 cells (D) by RT-qPCR. All the data are presented as the mean ± SEM of n=3 independent experiments. *P* values were calculated by two-sided unpaired Student's *t*-test; ns, not significant; ***P*<0.01; ****P*<0.001.

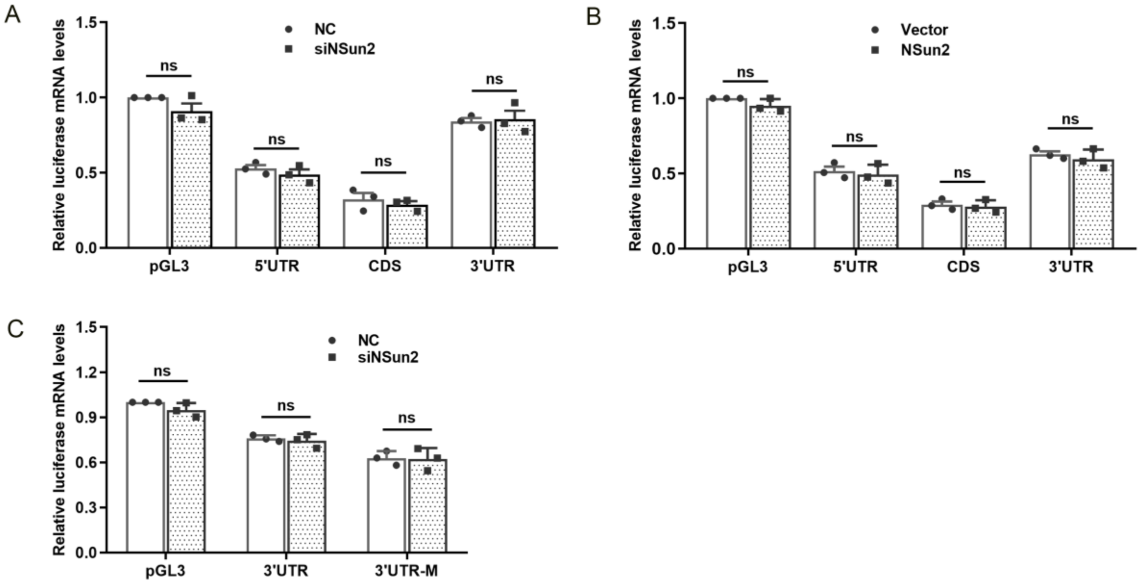

Figure S2. Effects of NSun2 on luciferase mRNA expression levels in HEK293T cells. (A) and (B) HEK293T cells were transfected with pGL3 luciferase reporter vector fused to the indicated ATX fragment for 24 h, and then the cells were transfected with NSun2 siRNA (A) or a plasmid expressing NSun2 (pcDNA3.1-NSun2) (B). At 48 h later, luciferase mRNA levels in the indicated cells were analyzed by RT-qPCR and normalized to Renilla mRNA levels. (C) The pGL3 luciferase reporter vector fused to ATX 3′UTR or 3′UTR-M (cytosine 2756 mutated to thymidine in 3′UTR) was transfected into HEK293T cells, which were then transfected with NC siRNA or NSun2 siRNA. Luciferase mRNA levels in each cell were detected at 48 h after siRNA transfection. All the data are presented as the mean ± SEM of n=3 independent experiments. *P* values were calculated by two-sided unpaired Student's *t*-test; ns, not significant.

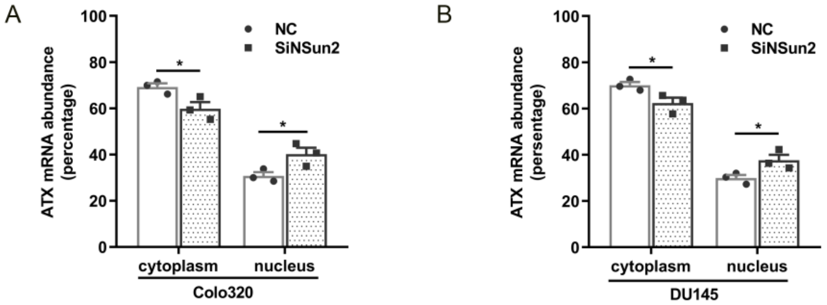

Figure S3. Effects of NSun2 on the nuclear export of ATX mRNA in Colo320 and DU145 cells. Colo320 and DU145 cells were treated with the NC or NSun2 siRNA as indicated. The percentage content of cytoplasmic and nuclear ATX mRNA in the indicated Colo320 cells (A) and DU145 cells (B) were analyzed by RT-qPCR. The RT-qPCR data were presented as the mean ± SEM of the results of 3 independent experiments. *P* values were calculated by two-sided unpaired Student's *t*-test; ns, not significant; **P*<0.05.

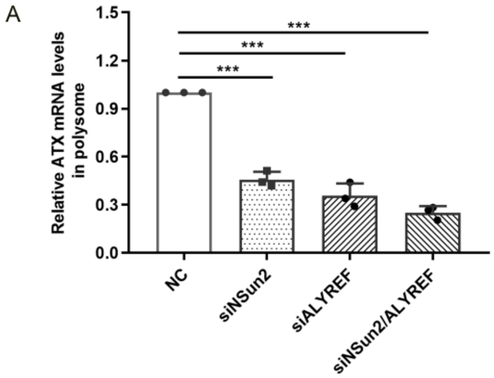

Figure S4. Knockdown of NSun2 and ALYREF decreased the recruitment of ATX mRNA to polysome. (A) U87 cells transfected with NC siRNA, NSun2 siRNA, ALYREF siRNA or both NSun2 and ALYREF siRNAs for 48 h were used to isolate the polysomal and nonpolysomal fractions. RNA was extracted from the polysomal fraction, and RT-qPCR analysis was performed to measure the content of ATX mRNA in polysomal fraction. All the data are presented as the mean ± SEM of n=3 independent experiments. *P* values were calculated by two-sided unpaired Student's *t*-test; ****P*<0.001.

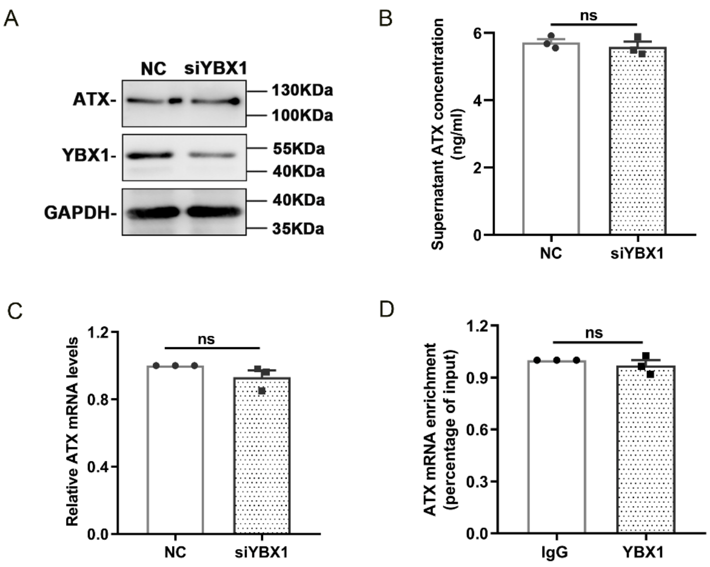

Figure S5. Effects of m^{5}C reader YBX1 on ATX expression in U87 cells. U87 cells were treated with NC or YBX1 siRNA as indicated. (A) and (B) ATX protein levels in cell culture medium and YBX1 protein levels in cells were tested by Western blot (A), while ATX concentration in cell culture medium was further assessed via a human ATX ELISA kit (B). (C) ATX mRNA levels were assessed in the indicated U87 cells by RT-qPCR. (D) UV crosslink RNP IP assays to detect the binding of endogenous YBX1 protein to ATX mRNA in U87 cells. All the data are presented as the mean ± SEM of n=3 independent experiments. *P* values were calculated by two-sided unpaired Student's *t*-test; ns, not significant.
